# Absences due to illness and health problems of the teachers of municipal public schools in Rio Branco, Acre, Brazil

**DOI:** 10.47626/1679-4435-2023-1094

**Published:** 2023-11-24

**Authors:** Elivan da Silva Dias, Sabrina da Silva Santos

**Affiliations:** 1 Programa de Pós-Graduação em Saúde Coletiva, Universidade Federal do Acre, Rio Branco, AC, Brazil; 2 Escola Nacional de Saúde Pública Sérgio Arouca, Fundação Oswaldo Cruz, Rio de Janeiro, RJ, Brazil

**Keywords:** schoolteachers, absenteeism, occupational health, docentes, absenteísmo, saúde do trabalhador

## Abstract

**Introduction:**

Absenteeism is defined as the absence of workers from their workplace, resulting from a complex phenomenon.

**Objectives:**

To analyze the profile of absence due to illness and health problems of the permanent teachers of early childhood education (day care and preschool) and elementary school (years 1-5) in the city of Rio Branco - Acre, Brazil, from 2014 to 2017.

**Methods:**

An observational, analytical, individual, cross-sectional, retrospective cohort study with data from 1,584 teachers from 100 municipal public schools in Rio Branco. The period prevalence was calculated by chapters of the International Classification of Diseases, 10th revision, and by specific reasons for absence for those chapters with higher proportions. Additionally, the absenteeism-illness frequency index and mean sickness absence duration were calculated.

**Results:**

The chapters of the International Classification of Diseases, 10th revision, with the highest prevalence of absences were Chapter XIII (diseases of the musculoskeletal system and connective tissue) with 7.83%, Chapter V (mental and behavioral disorders) with 7.45%, and Chapter X (diseases of the respiratory system) with 6.12%. The absenteeism-illness frequency index was 0.72, and the mean sickness absence duration was 24.07 days.

**Conclusions:**

The present study showed that diseases of the musculoskeletal system and connective tissue, mental and behavioral disorders, and diseases of the respiratory system are the main problems that lead the permanent teachers of municipal public schools in Rio Branco to be absent from the classroom.

## Abstract (pt)

**Introdução:**

Absenteísmo é definido como a ausência do trabalhador no seu local de trabalho, sendo resultado de um fenômeno complexo.

**Objetivos:**

Analisar o perfil de afastamento por motivo de doenças e agravos de saúde dos professores efetivos da educação infantil (creche e pré-escola) e ensino fundamental I (1º ao 5º ano) do município de Rio Branco - Acre, de 2014 a 2017.

**Métodos:**

Estudo observacional, analítico, individual, transversal, retrospectivo de coorte com dados de 1.584 professores das 100 escolas municipais de Rio Branco. Foram calculadas as prevalências de período por capítulos da Classificação Internacional de Doenças, 10ª revisão, e por motivos específicos de afastamentos para aqueles capítulos com maiores proporções. Adicionalmente, foram calculados o índice de frequência de absenteísmo-doença e a duração média das ausências.

**Resultados:**

Os capítulos da Classificação Internacional de Doenças, 10ª revisão, com as maiores prevalências de afastamento foram: Capítulo XIII (doenças do sistema osteomuscular e do tecido conjuntivo) com 7,83%; Capítulo V (transtornos mentais e comportamentais) com 7,45%; e Capítulo X (doenças do aparelho respiratório) com 6,12%. O índice de frequência de absenteísmo-doença foi de 0,72 e a duração média dos afastamentos foi de 24,07 dias.

**Conclusões:**

O presente estudo evidenciou que as doenças osteomusculares e conjuntivas, os transtornos mentais e comportamentais e as doenças respiratórias são os principais agravos que levam os professores efetivos das escolas públicas municipais de Rio Branco a se ausentarem da sala de aula.

## INTRODUCTION

Absenteeism is defined as the absence of workers from their workplace resulting from a complex phenomenon, which can be caused by several factors. This absence of workers may be related to working conditions, such as poor hygiene and safety conditions, which, in turn, may lead to occupational accidents and workers’ dissatisfaction with the sociocultural reality and with factors related to the worker’s personality and health conditions. Regardless of the causative reason, absences from work entail several problems for professional organizations as a whole.^1^

Regarding teacher absenteeism, the consequences can be devastating for students during the teaching-learning process, leading to incalculable long-term damage. Additionally, when analyzing this issue, it is of utmost importance to point out that factors related to routine, work dynamics, and physical-structural conditions of schools, among others, contribute to teachers’ dissatisfaction with the organization and with the work performed, leading, directly or indirectly, to an increase in absenteeism.^2^

Teacher absenteeism due to illness and health problems, in turn, has been shown to be a public health problem - especially when one considers that, at least in part, these absences are due to unsatisfactory working conditions, work overload, and violence in the school environment, among other stressors that can lead to illness, loss of quality of life, and even permanent damage to the teacher’s health.^3^

Studies to characterize the health of Brazilian teachers are scarce, and if we consider the reality of the North region of Brazil, this production is virtually non-existent.^4,5^ Published studies report on the sources and symptoms of occupational stress, highlighting the precariousness of working conditions as the main cause of physical and psychological distress among teachers.^6^

Therefore, the present study aimed to describe the profile of absence due to illness and health problems of education workers (teachers) in the city of Rio Branco, state of Acre, Brazil, from 2014 to 2017.

## METHODS

This is a cross-sectional descriptive epidemiological study of permanent teachers who worked from 2014 to 2017 in 100 municipal public schools of early childhood education (day care and preschool) and elementary school (years 1-5) in urban and rural areas of the city of Rio Branco.

The study population consisted of the universe of teachers from the permanent staff of the Municipal Department of Education (Secretaria Municipal de Educação [SEME]) of Rio Branco who worked in early childhood education (day care and preschool) and elementary school (years 1-5) from 2014 to 2017. This study period was defined based on the fact that SEME human resources started the digital tabulation of these data only in 2014. Temporary contract teachers were not included in the study because SEME does not hold the employee folder’s files of these workers.

All medical certificates (up to 15 days of sick leave) and medical leaves (more than 15 days of leave with referral to a social security medical expert) recorded at the SEME Human Resources Division from January 1, 2014, to December 31, 2017, were reviewed to identify the reasons for absenteeism using the International Classification of Diseases, 10th revision (ICD-10), as a reference and to calculate sickness absence duration. Of note, employees in Brazil are entitled to 15 days of sick leave paid by employers, and medical certification of illness is required in these cases for sick leave to be granted. After 15 days, social security pays for sickness benefits to insured employees as long as the illness lasts, and medical examination by a social security medical expert for certification of illness is required in these cases for sickness benefits to be granted. Absences unrelated to the health condition of permanent teachers, such as maternity leave and leave to attend medical appointments with their children, were not included in the analysis.

The period prevalence was calculated (number of teachers on leave/total number of teachers from 2014 to 2017) by ICD-10 chapters and by specific reasons for absence for those chapters with higher proportions.

Additionally, two absenteeism-illness indicators proposed by Hensing et al.^7^ were calculated, namely: the absenteeism-illness frequency index (number of absence events/population at risk) and mean sickness absence duration (number of days absent/number of absence events).

In compliance with Resolution 466/12 of the National Health Council (Conselho Nacional de Saúde, Brazil, 1996), which provides for guidelines and regulatory standards for research involving human subjects, the present project was approved by the Research Ethics Committee of the Universidade Federal do Acre, under CAAE number 11099019.8.0000.5010.

## RESULTS

The analyzed population consisted of 1,584 permanent teachers who worked in basic education from 2014 to 2017. Of these, 1,039 worked in 2014, 971 in 2015, 997 in 2016, and 1,338 in 2017.

Table 1 shows the sociodemographic characteristics and work-related variables of the permanent teachers of municipal public schools from 2014 to 2017. There was a predominance of female teachers (84.4%), working in elementary school (years 1-5) (57.8%), working in schools in the urban area of Rio Branco (95.2%), and with 11 or more years of service at the beginning of the study (January 1, 2014) (43.7%). Regarding age, most teachers were aged 30 to 39 years (35%), with a minimum age of 23 years and a maximum age of 67 years (mean of 40 years). In addition, 85% of teachers worked 25 hours per week in the municipal public schools. These teachers had only one employment contract for city-level public education and could work in preschool or elementary school. Teachers working 40 hours per week were those who worked in public day care centers. Teachers with two employment contracts (permanent or supplementary working hours) in the municipal public schools were classified as the group working 50 hours per week, being able to work in preschool or elementary school.

**Table 1:** Sociodemographic characteristics and work-related variables of the permanent teachers of municipal public schools in Rio Branco, state of Acre, Brazil, 2014-2017

Variables/categories	n	%
Sex		
Male	241	15.2
Female	1,343	84.8
Age (years)		
≤29	248	15.7
30 to 39	554	35.0
40 to 49	470	29.7
50 to 59	246	15.5
60 or more	66	4.2
Working hours in municipal schools (hours)		
25	1,347	85.0
40	182	11.5
50 (dual employment)	55	3.5
Stages		
Day care	182	11.5
Preschool	486	30.7
Elementary school	916	57.8
Location		
Urban area	1,508	95.2
Rural area	76	4.8
Length of service on 01/01/2014 (years)		
Zero	494	31.2
Less than 1 year	160	10.1
1-10 years	238	15.0
11 or more years	692	43.7

During the study period, 471 permanent teachers submitted 1,151 sickness absences to the SEME Human Resources Division, 203 of which were medical leaves with absence exceeding 15 days and 948 were medical certificates with up to 15 days absent from work. The period prevalence of sickness absence for the total number of permanent teachers was 29.73%. Regarding the stage of education, day care teachers had the highest period prevalence (46.15%), followed by preschool (30.65%) and elementary school teachers (27.51%). The period prevalence for 3 or more absence events was 10.10%. The day care group also had a higher period prevalence for 3 or more absence events (14.28%) than preschool (11.52%) and elementary school teachers (8.51%) (Table 2).

**Table 2:** Profile of absence due to illness and health problems of the permanent teachers of municipal public schools in Rio Branco, state of Acre, Brazil, 2014-2017

Indicators	Day care (n = 182)	Preschool (n = 486)	Elementary school (n = 916)	Total (n = 1,584)
No. of teachers on leave	84	149	252	471
Period prevalence^*^ (1 absence event)	46.15	30.65	27.51	29.73
No. of teachers with 3 or more absence events	26	56	78	160
Period prevalence^*^ (3 or more absence events)	14.28	11.52	8.51	10.10
No. of medical leaves (≥ 15 days)	11	62	130	203
No. of medical certificates (< 15 days)	173	310	465	948
Total absence events	184	372	595	1,151
Absenteeism-illness frequency index†	1.01	0.76	0.64	0.72
No. of days absent	1,986	8,132	17,587	27,705
Mean sickness absence duration‡	10.79	21.86	29.56	24.07

* Number of teachers on leave/total number of teachers from 2014 to 2017.

† Number of absence events/number of teachers at risk.

‡ Number of days absent/number of absence events.

Table 2 shows the absenteeism-illness frequency index of the permanent teachers of municipal public schools, which was calculated by dividing the number of absence events by the number of teachers at risk of leaving due to illness and health problems in each stage of education. The overall absenteeism-illness frequency index was 0.72. In the analysis by stage of education, this index was higher in day care teachers (1.01) with 184 absence events, followed by preschool teachers (0.76) with 372 absence events and elementary school teachers (0.64) with 595 absence events.

The mean sickness absence duration was 24.07 days. The longest mean sickness absence duration was observed among elementary school teachers (mean of 29.56 days), followed by preschool teachers (mean of 21.86 days) and day care teachers (mean of 10.79 days).

The medical certificates and medical leaves presented by the permanent teachers of municipal public schools in Rio Branco, from 2014 to 2017, fit into 18 of the 21 ICD-10 chapters (Table 3). No teacher had absences related to ICD-10 chapters XVI (certain conditions originating in the perinatal period), XVII (congenital malformations, deformations and chromosomal abnormalities), or XX (external causes of morbidity and mortality).

**Table 3:** Number of absence events, proportion among total number of absence events, and period prevalence by chapters of the International Classification of Diseases, 10th revision (ICD-10), of the permanent teachers of municipal public schools in Rio Branco, state of Acre, Brazil, 2014-2017

ICD-10 chapter	No. of medical leaves (≥15 days)	No. of medical certificates (<15 days)	No. of absence events	Proportion (%)	Prevalence (%)
XIII - Diseases of the musculoskeletal system and connective tissue	62	213	275	23.89	7.83
V - Mental and behavioral disorders	67	225	292	25.36	7.45
X - Diseases of the respiratory system	8	183	191	16.59	6.12
IX - Diseases of the circulatory system	14	59	73	6.34	2.46
XXI - Factors influencing health status and contact with health services	11	22	33	2.86	1.83
VI - Diseases of the nervous system	2	49	51	4.43	1.52
XI - Diseases of the digestive system	3	34	37	3.21	1.20
XIX - Injury, poisoning and certain other consequences of external causes	13	26	39	3.38	1.14
XVIII - Symptoms, signs and abnormal clinical and laboratory findings, not elsewhere classified	9	14	23	1.99	1.01
XIV - Diseases of the genitourinary system	9	15	24	2.08	0.88
IV - Endocrine, nutritional and metabolic diseases	2	20	22	1.91	0.88
I - Certain infectious and parasitic diseases	3	18	21	1.82	0.88
VII - Diseases of the eye and adnexa	0	14	14	1.21	0.57
No ICD code	0	13	13	1.29	0.38
II - Neoplasms	4	5	9	0.78	0.38
XV - Pregnancy, childbirth and puerperium	0	9	9	0.78	0.25
VIII - Diseases of the ear and mastoid process	4	2	6	0.52	0.25
III - Diseases of the blood and blood-forming organs and certain disorders involving the immune mechanism	2	0	2	0.17	0.06
XII - Diseases of the skin and subcutaneous tissue	0	1	1	0.08	0.06

The ICD-10 chapters with the highest prevalence of absences were Chapter XIII (diseases of the musculoskeletal system and connective tissue) with 7.83%, Chapter V (mental and behavioral disorders) with 7.45%, and Chapter X (diseases of the respiratory system) with 6.12%. However, mental and behavioral disorders were associated with the highest number of absence events: 292 (25.36% of the absences recorded).

Table 4 shows that, for the study period, the permanent teachers of municipal public schools were absent from work for 29,420 days, adding up the days of sickness absence on medical certificates (< 15 days) and on medical leaves (≥ 15 days). When grouping the days absent from work according to the ICD-10 chapters, the highest proportion of days absent was observed in Chapter XIII - Diseases of the musculoskeletal system and connective tissue (Codes M00 - M99), accounting for 26.89% of days absent from work. Mental and behavioral disorders (Chapter V, Codes F00 - F99) led teachers to be absent from the classroom for 7,696 days, accounting for 26.16% of total days absent. Diseases of the respiratory system (Chapter X, Codes J00 - J99) had a marked participation in the number of days teachers were absent from work, accounting for 9.46% of the total number of days absent.

**Table 4:** Days absent from work by chapters of the International Classification of Diseases, 10th revision (ICD-10), of the permanent teachers of municipal public schools in Rio Branco, state of Acre, Brazil, 2014-2017

ICD-10 chapter	Total days of medical leave (≥15 days)	Total days of medical certificate (<15 days)	Total days absent	Proportion of days absent (%)
XIII - Diseases of the musculoskeletal system and connective tissue	5,969	1,941	7,910	26.89
V - Mental and behavioral disorders	5,676	2,020	7,696	26.16
X - Diseases of the respiratory system	1,166	1,616	2,782	9.46
XIV - Diseases of the genitourinary system	1,973	97	2,070	7.04
IX - Diseases of the circulatory system	1,346	421	1,767	6.01
XIX - Injury, poisoning and certain other consequences of external causes	1,458	214	1,672	5.68
XXI - Factors influencing health status and contact with health services	768	206	974	3.31
XVII - Symptoms, signs and abnormal clinical and laboratory findings, not elsewhere classified	848	88	936	3.18
II - Neoplasms	840	9	849	2.89
VI - Diseases of the nervous system	368	304	672	2.28
I - Certain infectious and parasitic diseases	451	120	571	1.94
XI - Diseases of the digestive system	180	247	427	1.45
IV - Endocrine, nutritional and metabolic diseases	226	167	393	1.24
VIII - Diseases of the ear and mastoid process	185	30	215	0.73
III - Diseases of the blood and blood-forming organs and certain disorders involving the immune mechanism	210	0	210	0.71
No ICD code	0	131	131	0.45
XV - Pregnancy, childbirth and puerperium	0	86	86	0.29
VII - Diseases of the eye and adnexa	0	58	58	0.20
XII - Diseases of the skin and subcutaneous tissue	0	1	1	0.00
Total	21,664	7,756	29,420	100.00

Table 5 shows the data on sickness absence for the top 10 specific causes from ICD-10 chapters XIII, V, and X. Regarding Chapter XIII, the highest proportion of absence events was due to dorsalgia (M54), accounting for 8.43% of the total number of all-cause absences submitted to the SEME Human Resources Division, with a period prevalence of 3.16%, leaving teachers out of the classroom for a total of 1,814 days. Intervertebral disc disorders (M51) also showed a high proportion (4.43%) and period prevalence (1.33%), making teachers absent from work for 1,776 days. Shoulder lesions (M75) appeared in third position, accounting for 1.43% of all-cause absences with a period prevalence of 0.63%, leading to 851 days of teacher absenteeism.

**Table 5:** Number of absence events, proportion among total number of all-cause absence events, period prevalence, and days absent from work for the top 10 specific causes from Chapters XIII, V, and X of the International Classification of Diseases, 10th revision (ICD-10), of the permanent teachers of municipal public schools in Rio Branco, state of Acre, Brazil, 2014-2017

ICD-10	Absence events	Proportion (%)	Prevalence (%)	Days
Chapter XIII - Diseases of the musculoskeletal system and connective tissue (M00 - M99)				
M54 - Dorsalgia	97	8.43	3.16	1,814
M51 - Other intervertebral disc disorders	51	4.43	1.33	1,776
M75 - Shoulder lesions	21	1.82	0.63	851
M50 - Cervical disc disorders	15	1.30	0.32	284
M79 - Other soft tissue disorders, not elsewhere classified	14	1.22	0.51	435
M41 - Scoliosis	12	1.04	0.32	68
M17 - Gonarthrosis [knee arthrosis]	10	0.87	0.32	720
M19 - Other arthrosis	9	0.78	0.25	211
M65 - Synovitis and tenosynovitis	8	0.70	0.32	317
M06 - Other rheumatoid arthritis	5	0.43	0.19	189
Chapter V - Mental and behavioral disorders (F00 - F99)				
F32 - Depressive episode	113	9.82	2.90	2,895
F41- Other anxiety disorders	98	8.51	2.21	1,236
F31 - Bipolar affective disorder	35	3.04	0.82	2,851
F33 - Recurrent depressive disorder	32	2.78	1.20	584
F25 - Schizoaffective disorders	4	0.35	0.06	30
F28 - Other nonorganic psychotic disorders	2	0.17	0.06	16
F40 - Phobic anxiety disorders	2	0.17	0.13	30
F42 - Obsessive-compulsive disorder	2	0.17	0.06	6
F04 - Organic amnesic syndrome, not induced by alcohol and other psychoactive substances	1	0.09	0.06	15
F10 - Mental and behavioral disorders due to use of alcohol	1	0.09	0.06	2
Chapter X - Diseases of the respiratory system (J00 - J99)				
J38 - Diseases of vocal cords and larynx, not elsewhere classified	41	3.56	1.26	760
J00 - Acute nasopharyngitis (common cold)	27	2.35	0.82	205
J03 - Acute tonsillitis	23	2.00	0.82	90
J45 - Asthma	18	1.56	0.88	790
J11 - Influenza, virus not identified	17	1.48	0.82	142
J01 - Acute sinusitis	11	0.96	0.32	61
J37 - Chronic laryngitis and laryngotracheitis	9	0.78	0.25	75
J35 - Chronic diseases of tonsils and adenoids	7	0.61	0.25	308
J02 - Acute pharyngitis, unspecified	6	0.52	0.19	58
J15 - Bacterial pneumonia, not elsewhere classified	5	0.43	0.13	41

Regarding Chapter V, the main reason for absences was depressive episode (F32), leaving teachers out of the classroom for 2,895 days, accounting for 9.82% of all-cause absences with a period prevalence of 2.92%. Anxiety disorders (F41) were the second leading cause of teacher absenteeism (8.41% of all-cause absences), with a period prevalence of 2.21%, leaving teachers out of the classroom for 1,236 days. Bipolar affective disorder (F31) had a marked participation in the number of days teachers were absent from work, accounting for 3.04% of all-cause absences with a period prevalence of 0.82%.

Regarding Chapter X, teachers were absent from the classroom for 760 days due to diseases of vocal cords and larynx (J38), accounting for 3.56% of all-cause absences with a period prevalence of 1.26%. Common cold (J00 - acute nasopharyngitis) was the second leading cause of sickness absence, accounting for 2.35% of all-cause absences with a period prevalence of 0.82%. Acute tonsillitis (J03) kept teachers out of the classroom for 90 days, accounting for 2.00% of all-cause absences with a period prevalence of 0.82%.

## DISCUSSION

The analysis of absences due to illness and health problems of 1,584 permanent teachers from municipal schools in the city of Rio Branco, between 2014 and 2017, showed that the highest proportions regarding the number of days absent from work were those related to diseases of the musculoskeletal system and connective tissue (ICD-10 Chapter XIII; 26.89% of days absent), followed by mental and behavioral disorders (ICD-10 Chapter V; 26.16%) and diseases of the respiratory system (ICD-10 Chapter X; 9.46%).

The diseases of public school teachers (mainly in basic education) in Argentina, Chile, Ecuador, Mexico, Peru, and Uruguay follow a pathological profile similar to that identified in the present study, according to data from a cross-sectional analysis conducted by the Regional Bureau for Education in Latin America and the Caribbean (Oficina Regional de Educación de la UNESCO para América Latina y el Caribe [OREALC/UNESCO]). Through self-report interviews, the researchers classified the main diseases of teachers into 3 broad categories: health problems associated with ergonomic demands; mental health problems; and general health problems, among which seasonal diseases and chronic diseases stand out as relevant.^3^

In the present study, ICD-10 Chapter XIII (diseases of the musculoskeletal system and connective tissue) had the reasons that left teachers out of the classroom for 7,910 days (275 absence events; period prevalence of 7.83%). The physical effort characteristic of teaching, which involves repetitive activities often performed in ergonomically inappropriate environments, is an important factor that, added to individual lifestyle characteristics, forms an interconnected network of factors that can help explain the development of the aforementioned diseases of the musculoskeletal system and connective tissue in teachers.^8^

The study conducted by Fernandes et al.,^9^ which aimed to determine the impact of musculoskeletal symptoms on the quality of life of the teachers of municipal public schools in the city of Natal, state of Rio Grande do Norte, showed that 47.7% of the interviewees reported being unable to perform their tasks due to symptoms in the previous 12 months. In the same vein, the survey conducted by Branco & Jansen^10^ pointed out that 36.6% of the teachers could not perform the activities they used to do previously. A cross-sectional study conducted in Ethiopia between 2016 and 2017 involving 754 secondary school teachers showed a self-reported prevalence of 57.3% of shoulder and neck injuries.^11^

In the present study, of the diseases listed in ICD-10 Chapter XIII, the one with the highest proportion of absences was dorsalgia (M54), with 8.43% of absence events, followed by other intervertebral disc disorders (M51), with 4.43%, and shoulder lesions (M75), with 1.82%. These problems may be related to standing posture and spinal movements (inclination or rotation) during work, which can increase internal pressure on intervertebral discs and lead to severe pain and even paralysis.^12^ The standing posture creates an overload on the body supporting structures, which increases the load on the lumbosacral region and may cause a herniated disc, for example. Keeping the shoulders in the same position for a long time may cause inflammatory processes such as tendinitis and bursitis, leading to ligament and joint impairment.^13^

Mental and behavioral disorders (ICD-10 Chapter V), which had a period prevalence of 7.45% in this study, with 7,696 days absent from work (292 absences), are characterized as conditions in which changes in thinking and mood (emotions) are present, as well as behaviors associated with personal distress and/or deterioration of psychic functioning.^14^ Studies suggest that the high proportions of absences due to mental and behavioral disorders may be associated with the high psychological demand required in the performance of teaching activities, poor control over their own work tasks, longer time in the profession, high weekly working hours, multiple jobs, and a series of characteristics related to the teaching environment and work organization, such as work pace, environment in inadequate conditions, and stressful interpersonal relationships, among other factors.^15,16^

A review of the international literature conducted by Scheuch et al.^17^ suggests that mental and psychosomatic diseases are more common in teachers than in other professional groups, as are nonspecific complaints such as exhaustion, fatigue, headache, and tension. Additionally, it is estimated that 3%-5% of teachers suffer from burnout, but these data may vary according to the definition of the term adopted.^17^

In the state of Rio Grande do Sul, in the city of Pelotas, a survey conducted with all teachers working in municipal public schools had as one of its objectives to analyze the teaching work process and the repercussions on teachers’ health. The results of this study showed that early childhood teachers were the ones who most frequently requested medical leave, with mental problems appearing in first position, followed by behavioral problems and diseases of the musculoskeletal system.^18^ A cross-sectional study of 751 elementary school teachers from municipal public schools in the city of Belo Horizonte, state of Minas Gerais, showed a prevalence of mental and behavioral disorders of 50.3%.^19^ Likewise, the study conducted by Bannai et al.^20^ including 522 teachers in the province of Hokkaido, Japan, identified the presence of psychological distress in 47.8% of men and 57.8% of women.

In the present study, of the diseases listed in ICD-10 Chapter V, the one with the highest proportion of absences was depressive episode (F32), with 9.82% of absence events, followed by other anxiety disorders (F41), with 8.51%, and bipolar affective disorder (F31), with 3.04%. According to the Brazilian Ministry of Health,^14^ these disorders are classified into mood/affective disorders (F30-F39) and neurotic, stress-related disorders and somatoform disorders (F40-F48).

Diseases of the respiratory system (ICD-10 Chapter X) also showed a high period prevalence (6.12%), leaving teachers in Rio Branco out of the classroom for 2,782 days (191 absences). These diseases range from a common cold to chronic problems such as tonsillitis, laryngitis, pharyngitis, and vocal cord injuries.^21^ Suboptimal environmental conditions in schools, in terms of noise levels, cleanliness, ventilation, lighting, temperature, and large number of students per classroom, added to excessive activities and inappropriate resting, make teachers a high-risk group for voice disorders, with consequent absenteeism, requiring readaptation to work.^22^

A cross-sectional study entitled “Work absenteeism due to voice disorders in Brazilian schoolteachers,” including 6,510 teachers between 2015 and 2016, identified that the main health problem that kept teachers out of the classroom, for short periods (up to 5 days), was voice disorder (17.7%), followed by reports of respiratory problems (14.6%).^23^ International studies have also reported the presence of voice disorders in teachers. A survey conducted with 1,617 public primary and secondary school teachers in South Korea revealed a prevalence of voice disorders of 13.1% in teachers working in the classroom and of 8% in teachers performing other tasks.^24^ In Germany, of 536 teachers from early childhood education, elementary schools, high schools, special schools, and vocational schools analyzed, 58.3% reported having experienced a voice problem.^25^

In the present study, of the diseases listed in ICD-10 Chapter X, the one with the highest proportion of absences was diseases of vocal cords and larynx, not elsewhere classified (J38), with 3.56% of absence events, followed by acute nasopharyngitis (common cold - J00), with 2.35%, and acute tonsillitis (J03), with 2.00%. Ferreira et al.^26^ state that the characteristics of teaching, previously mentioned, are associated with hoarseness, vocal fatigue, sore throat, effort to speak, difficulty maintaining the intensity of vocalizations, and aphonia.

Absenteeism-illness indicators, such as the absenteeism-illness frequency index and the mean sickness absence duration used in the present study, are internationally accepted parameters that aim to measure absence from the workplace due to illness, allowing us to monitor variations over a period and to compare results within and between groups of workers. The analysis of these indicators highlights not only the epidemiological situation of workers but also their working conditions, providing elements for planning actions aimed at workers’ health, as well as their effectiveness.^27^

The permanent teachers of municipal public schools in Rio Branco had 1,151 absence events between 2014 and 2017, for an absenteeism-illness frequency index of 0.72. Considering the stage of education, the index was higher in day care teachers (1.01), followed by preschool teachers (0.76) and elementary school teachers (0.64). The mean sickness absence duration was 24.07 days, with elementary school teachers having the longest mean duration (29.56 days), followed by preschool teachers (21.56 days) and day care teachers (10.79 days).

The absenteeism-illness frequency indices in this study were lower than those found in the study by Daniel et al.,^28^ who analyzed municipal civil servants in the municipal government of Curitiba, state of Paraná, from 2010 to 2015, and reported an absenteeism-illness frequency index of 2.03 in 2015, and a mean sickness absence duration of 6.58 days.

Santos & Mattos^29^ conducted a survey on absenteeism-illness in the municipal government of Porto Alegre, state of Rio Grande do Sul, aiming to analyze the medical leaves of municipal civil servants between 2004 and 2005. The highest absenteeism-illness frequency index was presented by the department of education, accounting for 4.8% of the absence events during the study period.

The present study has some limitations, mainly inherent to its descriptive design, making it impossible to establish causal relationships. The analyzed data do not allow distinguishing occupational morbidities from common diseases, thus precluding a proper analysis of the impact of the teaching job on the teachers’ illness profile. Furthermore, only the permanent teachers of municipal public schools in Rio Branco were considered for inclusion during the study period due to the unavailability of the sick leave files of temporary contract teachers. Another limitation is that this study does not report whether the teachers working in the municipal public schools had another employment contract as a teacher in a private school or in a state-run public school. Hirata et al.,^30^ analyzing data from the National Institute for Educational Studies and Research Anísio Teixeira (Inep) in 2017, investigated the rate of teachers working one or more shifts in basic education. The authors reported a rate of 87.8% of early childhood teachers and 80.5% of elementary school teachers working only one shift. Therefore, it is possible that the percentage of teachers working in each of the shifts in our municipal schools is very close to the actual percentage of working hours of these professionals.

Despite these limitations, the present study has strengths such as the uniqueness of the data used, which correspond to the universe of schools and teachers from the permanent staff of the SEME in Rio Branco during the study period, thus avoiding problems related to sampling, which are common in smaller studies. In addition, this study presents a 4-year data analysis, allowing a more homogeneous and accurate evaluation by reducing possible annual oscillations.

## CONCLUSIONS

The results obtained in this study indicated a high prevalence of periods of sickness absence for teachers working in municipal public schools in Rio Branco related to diseases of the musculoskeletal system and connective tissue, mental and behavioral disorders, and diseases of the respiratory system. Conducting further studies with other designs is important to clarify the factors associated with this illness among teachers, thus contributing to a better understanding of the health of this population and to the implementation of health promotion strategies for teachers.
